# Osteoblasts sense extracellular levels of phosphate to control the local expression of phosphatases for matrix mineralisation

**DOI:** 10.1016/j.bonr.2025.101863

**Published:** 2025-07-30

**Authors:** Soher Nagi Jayash, Thomas Duff, Qaisar Tanveer, Worachet Promruk, Colin Farquharson

**Affiliations:** The Roslin Institute and Royal (Dick) School of Veterinary Studies, University of Edinburgh, Easter Bush Campus, Midlothian EH25 9RG, Scotland, UK

**Keywords:** Osteoblast, Phosphate, Mineralisation, PHOSPHO1, Tissue-nonspecific alkaline phosphatase, Type III Na- P_i_ co-transporters, Fibroblast growth factor receptor

## Abstract

The provision of inorganic phosphate (P_i_) for biomineralisation is under systemic and local control. Locally, osteoblast production of phosphatases such as tissue-nonspecific alkaline phosphatase (TNAP) and PHOSPHO1 is required for normal skeletal mineralisation and osteoblasts may sense extracellular P_i_ concentrations to control local phosphatase activity and thereby “fine-tune” P_i_ production and delivery for biomineralisation. This has been poorly explored and this study examined the ability of osteoblasts to sense and respond to extracellular P_i_ to control the local expression of TNAP and PHOSPHO1.

Extracellular P_i_ downregulated the expression of PHOSPHO1 and TNAP by human primary osteoblasts at both mRNA and protein levels. Increasing P_i_ concentrations also reduced the mRNA expression of the type III Na- P_i_ co-transporters, PiT-1 and PiT-2 and selectively enhanced ERK1/2 phosphorylation. Inhibition of PiT-1 and PiT-2 by Foscarnet or MEK1/2 by UO126 abolished the downregulation of *PHOSPHO1* and *ALPL* expression by extracellular Pi. Moreover, extracellular P_i_ phosphorylated fibroblast growth factor receptor (FGFR) substrate 2α (FRS2α) and this activation was abolished by Foscarnet. Also, blocking FGFR signalling inhibited the phosphorylation of ERK1/2 and prevented the decrease in *ALPL* and *PHOSPHO1* expression by extracellular P_i_. Similar results were observed in cultured murine calvaria. Osteoblast matrix mineralisation by extracellular P_i_ was dependent upon type III Na- P_i_ co-transporters and FGFR signalling.

In conclusion, these results suggest an interplay between FGFR and P_i_ transporters is required for osteoblasts to sense and respond to extracellular P_i_. This understanding advances our knowledge of the molecular control of physiological bone mineralisation by osteoblasts.

## Highlights

• Extracellular Pi downregulates TNAP and PHOSPHO1 expression in human osteoblasts.
• Downregulation of phosphatase expression by extracellular Pi requires PiT1/2.
• ERK1/2 & FGFR signalling mediates extracellular Pi effects on phosphatase expression.
• Osteoblast mineralisation by extracellular Pi requires PiT1/2 & FGFR signalling.

## 1 Introduction

Phosphorous is indispensable for life and in its organic form it is required for a wide range of cellular functions such as membrane integrity (phospholipids), intracellular signalling (cAMP), metabolism (ATP) and nucleotide formation (RNA and DNA) (Peacock, 2021). In contrast, bone is the major reservoir for inorganic phosphate (P_i_) where approximately 85 % of Pi is stored as calcium phosphate crystals (Morgan et al., 2018). The amount of P_i_ within the circulation and the extracellular fluid of the bone matrix is tightly regulated and hypophosphatemia or impaired re-absorption of P_i_ by the kidney leads to defective mineralisation of collagen fibrils manifesting as rickets in growing children and osteomalacia in adults (Shimada et al., 2001; Carpenter et al., 2017). In contrast, in conditions such as chronic kidney disease, kidney function declines leading to the retention of P_i_ and the resulting hyperphosphatemia can result in ectopic calcification leading to cardiovascular morbidity and mortality (Fang et al., 2014).

Evidence from clinical conditions and genetically modified mice has also revealed that skeletal mineralisation is controlled by the local generation of P_i_ by phosphatases expressed by osteoblasts and chondrocytes. Specifically, the genetic disorder hypophosphatasia (HPP), a rare form of inherited rickets, osteomalacia and hypomineralisation of teeth, is caused by loss of function mutations in the alkaline phosphatase (*ALPL*) gene which encodes for the ectoenzyme tissue-nonspecific alkaline phosphatase (TNAP) (Millán and Whyte, 2016; Robison, 1923). In its absence or mutated form, TNAP is unable to hydrolyse a key inhibitor of mineralisation, inorganic pyrophosphate (PP_i_) and the attendant altered extracellular PP_i_/P_i_ ratio contributes to the defective mineralisation observed in HPP patients and in mice deficient in *Alpl* (Ciancaglini et al., 2010; Majeska and Wuthier, 1975). PHOSPHO1 (phosphoethanolamine/phosphocholine phosphatase 1), is expressed by both osteoblasts and chondrocytes and is essential for cartilage and bone mineralisation (Dillon et al., 2019). It generates P_i_ from membrane phospholipids such as phosphocholine and phosphoethanolamine and mice null for *Phospho1* display osteomalacia, spontaneous fractures, bowed long bones and scoliosis in early life (Huesa et al., 2011; Javaheri et al., 2015; Roberts et al., 2004; Yadav et al., 2011).

The contribution of extracellular P_i_ versus locally generated P_i_ to the mineralisation process remains obscure but skeletal cell culture studies have revealed that P_i_ regulates the expression of matrix proteins including the stimulation of recognised inhibitors of matrix mineralisation such as osteopontin (*Spp1*) and matrix Gla protein (*Mgp*) (Beck Jr. et al., 2000; Bon et al., 2018a; Camalier et al., 2013; Julien et al., 2009; Rendenbach et al., 2014). These data suggest a level of co-ordination between the osteoblast and its environment to deliver P_i_ to the matrix for hydroxyapatite formation. Our recent studies have shown that extracellular P_i_ decreases osteoblast PHOSPHO1 expression (Hsu et al., 2022) but the relationship between extracellular P_i_ and *Alpl* expression by osteoblasts is less clear (Camalier et al., 2013; Hsu et al., 2022; Orimo and Shimada, 2006). Nevertheless, the consensus of the available data infers that osteoblasts can sense extracellular P_i_ concentrations to control local phosphatase activity and fine-tune and/or rate-limit the availability of P_i_ for matrix mineralisation. This however has been poorly explored and the conflicting data from these previous studies may reflect the use of human and murine osteoblast-like cell lines (Camalier et al., 2013; Orimo and Shimada, 2006) Therefore, in this study we examined the ability of human primary osteoblasts to sense extracellular P_i_ concentrations to control the local expression of *ALPL* and *PHOSPHO1* and their translated protein.

## 2 Materials & methods

### 2.1 Laboratory consumables

All chemicals, materials and reagents were supplied by Sigma-Aldrich (Poole, UK) or less otherwise stated.

### 2.2 Osteoblast cell culture

In all cell culture experiments, normal human osteoblasts (NHOst) (Lonza, Basel, Switzerland) were used. These cells were obtained from the trabecular bone from various long bones of a single 33-year- old male donor and characterised by alkaline phosphatase expression and an ability to mineralise their matrix (Gunaratnam et al., 2013; Pearsall et al., 2008). Cryopreserved vials of were thawed and cultured in low glucose DMEM supplemented with 10 % foetal bovine serum (FBS) and 1 % penicillin-streptomycin (P/S) (all Gibco, Paisley, UK) until 80 % confluent. Thereafter, the cells were seeded at a density of 5000 cells/cm^2^ in multi-well plates and maintained in P_i_-free DMEM supplemented with 10 % FBS (United States Biological, Salem, MA, USA) and 1 % P/S in a humidified atmosphere containing 5 % CO₂ at 37 °C until confluent. In all studies, medium was changed every 2–3 days and the NHOst were used between passage 4 to 6. Although P_i_-free DMEM was used in all studies, we estimated that due to the presence of 10 % FBS this contributed 0.33 mM P_i_ to the basal culture medium (Lee et al., 2023). P_i_ in the form of NaH_2_PO_4_ was added to the basal culture medium at various concentrations and all mentions in the text and figures to the amount of P_i_ present in individual cultures refers to the amount of additional P_i_ added and does not include the 0.33 mM from the FBS present in the basal medium.

For intracellular signalling studies, the confluent cells were transferred to serum-free low glucose DMEM containing 0.1 % bovine serum albumin overnight. The following day, the cells were treated with fresh medium containing 5 mM P_i_ for 15, 30, 45, or 60 min. For positive controls, cells maintained in serum free low glucose DMEM were treated for 30 min with insulin (100 nM) to activate Erk1/2 and Akt, and anisomycin (2.5 μg/mL) to activate p38 and JNK. At the end of each timepoint, proteins were prepared for western blotting. The use of 5 mM P_i_ was based on previous studies where it was reported to elicit clear and robust differences in *Phospho1* and *Alpl* expression and activate the FGFR1-FRS2α-ERK pathway (Hsu et al., 2022; Takashi et al., 2019).

For all other studies, the confluent cultures were incubated in P_i_-free DMEM supplemented with 10 % FBS, 1 % P/S, 50 μg/mL l-ascorbic acid and 1.5 mM CaCl₂ for 10-days to promote the differentiated phenotype prior to Pi challenge ± inhibitors for 24 h. Supplementation with CaCl₂ promotes matrix mineralisation, negating the need for β-glycerophosphate which can enhance *ALPL* expression and introduce unwanted P_i_ into the culture medium (Chang et al., 2008; Huesa et al., 2015). To study the longer effects of Pi, cells were challenged with a defined concentration of P_i_ (final concentration 0–5 mM) for 2 weeks after confluency and RNA or protein was extracted and processed for RT-qPCR and western blotting, respectively. Inhibitors of MEK 1/2 (U0126; 30 μM; Cell Signalling Technology Europe, Leiden, Netherlands), fibroblast growth factor receptor (FGFR) (PD173074; 300 nM; Apexbio Technology, Houston Tx, USA) and type III Na- P_i_ co-transporters, PiT1 and PiT2 (phosphonoformic acid (Foscarnet); 300 μM) were introduced to the cultures 60 min prior to the addition of P_i_ to identify the intracellular signalling pathways involved in P_i_ sensing.

#### 2.2.1 Osteoblast matrix mineralisation

Confluent osteoblasts (in P_i_-free DMEM supplemented with 10 % FBS and 1 % P/S) were cultured for 2-weeks in P_i_ -free DMEM supplemented with 50 μg/mL ascorbic acid, 1.5 mM CaCl_2_ together with either 1) 0 mM P_i_, 2) 5 mM P_i_, 3) 5 mM P_i_ + 300 μM foscarnet, or 4) 5 mM P_i_ + 300 nM PD173074.

### 2.3 Animals and experimental protocols

#### 2.3.1 Mice

All animal studies were approved by the Roslin Institute's named veterinary surgeon and named animal care and welfare officer, with animals maintained in accordance with the Home Office code of practice (for the housing and care of animals bred, supplied or used for scientific purposes). Animal studies were conducted in line with the ARRIVE guidelines. Pregnant C57BL/6JCrl females were obtained from Charles River Laboratories (Margate, Kent, UK) and postnatal 3-day old pups (male and female) were culled by decapitation, calvaria removed and cut in half along the sagittal suture prior to culturing (Staines et al., 2019).

#### 2.3.2 Calvaria culture

The half-calvaria were cultured in a 24-well plate with 350 μL of pre-warmed P_i_ -free DMEM per well, supplemented with 10 % FBS and 1 % P/S, and incubated overnight. The following day, fresh medium supplemented with 50 μg/mL ascorbic acid and 1.5 mM CaCl₂ together with either 1) 0 mM P_i_, 2) 5 mM P_i_, 3) 5 mM P_i_ + 300 μM foscarnet, or 4) 5 mM P_i_ + 300 nM PD173074 was added the half-calvaria. In all cases, the calvariae were pre-incubated with foscarnet or PD173074 for 60 min prior to the addition of P_i_. Three replicates were used for each culture condition. After 3-days of culture, the half-calvaria were snap frozen in liquid nitrogen and stored at −80 °C for RNA extraction or stored in water at −20 °C for micro-CT scanning.

### 2.4 Micro-computed tomography (micro-CT) of calvariae

After 3-days of culture, high-resolution scans were acquired using a Neoscan80 micro-CT (Mechelen, Belgium). A pixel size of 5 μm was used with the following settings: 50 kV, 200 μA, no filter, and a 0.2° rotation angle. Four images were averaged at each rotation angle. Reconstructed images were generated using Skyscan NRecon software v1.6.9 (Bruker, Belgium), and bone volume was visualized and analyzed using CTAn software v1.20 (Skyscan). The whole calvaria half was selected for analysis. To evaluate bone mineral density (BMD), reference phantoms containing calcium hydroxyapatite at concentrations of 0.25 and 0.75 g/cm^3^ were scanned and reconstructed using identical imaging parameters as those used for the bone specimens. Baseline scans to assess within-sample changes were not done due to concerns with maintaining sterility and avoiding radiation damage to the calvaria.

### 2.5 Quantitative polymerase chain reaction (qPCR)

The NHOst were scraped in 250–500 μL QIAzol lysis reagent (Qiagen, Manchester, UK) and for calvaria, 1 mL of QIAzol was added and the tissue was homogenised by 3 × 10 s cycles of a Rotor-Stator tissue homogenizer (Ultra-Turrax T10). All samples were stored at −80 °C. Total RNA was extracted by adding chloroform at a volume equal to 0.2 times the total volume of the cell lysate and centrifuging at 12,000 x g for 15 min at 4 °C. The upper colourless phase was carefully aspirated into an Eppendorf tube and mixed with an equal volume of ethanol. RNA was purified using the Qiagen RNeasy® Mini Kit (Qiagen) according to the manufacturer's instructions, including an RNase-free DNase I (Qiagen) digestion step to eliminate genomic DNA contamination. The purified RNA was quantified using a NanoDrop spectrophotometer (ND-1000; Thermo Fisher Scientific, USA). For cDNA synthesis, 10 μL RNA samples were reverse transcribed using random primers (2 μL, 50 ng/μL) and loaded into a Dyad PCR system DNA engine Peltier thermal cycler (MJ Research), programmed at 70 °C for 10 min. A master mix was prepared by mixing 4 μL 5× First-Strand Buffer, 2 μL DTT, 1 μL dNTPs, and 1 μL Superscript II RNase H enzyme, and made up to 8 μL. This master mix (Life Technologies, Paisley, UK) was added to each RNA sample. The RNA samples were converted into cDNA using the following thermal profile in the Dyad PCR thermal cycler: 25 °C for 10 min; 42 °C for 50 min; 70 °C for 15 min, and held at 4 °C. The cDNA samples were then diluted to 5 ng/μL (25 ng in total) with RNase-free water and stored at −20 °C until use. qPCR was conducted to quantify gene expression levels. The setup included SYBR Green master mix (Primer Design, Eastleigh, UK) and specific primers (Thermo Fisher Scientific) (details provided in Supplementary Table 1). The master mix was prepared by combining 10 μL of SYBR Green, 1 μL of primer mix, and 4 μL of RNase-free water. To each reaction, 15 μL of the master mix was added to 5 μL of diluted cDNA. Gene expression analysis was then performed on a Stratagene Mx3000P real-time qPCR system (Agilent Technologies, USA) under the following conditions; 2 min at 95 °C (initial denaturing), 40 thermocycles consisting of 15 s at 94 °C (denaturing) and 1 min at 60 °C (annealing), the melt curve stage consisted of 1 min at 95 °C, 30 s at 55 °C (elongation) and 30s at 95 °C. The gene expression was normalized to the housekeeping genes *ATP5B* (human) and *Ppia* (mouse) and analyzed using the ΔΔCt method (Livak and Schmittgen, 2001).

### 2.6 Western blot analysis

Protein was extracted from NHOst cells using RIPA buffer containing a protease and phosphatase inhibitor cocktail (Thermo Fisher Scientific). The appropriate volume of protein lysate (20–40 μg) was determined using the bicinchoninic acid (BCA) protein assay kit (Thermo Fisher Scientific) and combined with 4× sample buffer and a reducing agent. The protein samples were denatured by heating at 95 °C for 8 min and then loaded onto a 4–12 % Bis-Tris protein gel (Thermo Fisher Scientific) and placed into a mini gel tank (Invitrogen, Paisley, UK) with MES-SDS running buffer. The gel was run for 90 min at 100 V using a Bio-Rad Power Pac300. Following electrophoretic separation, the proteins were transferred to a methanol-activated PVDF membrane using a semi-dry transfer method. This transfer was performed using a Bio-Rad Trans-Blot SD transfer cell for 60 min at 18 V, with the membrane sandwiched between transfer buffer-soaked extra thick filter paper. The membranes were blocked in LI-COR Odyssey Intercept (PBS) blocking buffer (LI-COR Biosciences, Chesterton, UK) for 1 h to prevent non-specific binding. Primary antibody incubation was carried out overnight at 4 °C to ensure maximum binding specificity and affinity. After primary antibody incubation, the membranes were incubated with secondary antibodies for 1 h. The membranes were then washed with PBS-Tween 20 and visualized using a LI-COR Odyssey infrared scanner (LI-COR Biosciences) with a resolution of 169 μm. Alternatively, the membranes were scanned with a GeneGnome imaging system (Syngene) according to the manufacturer's recommendations after being thoroughly wetted with the SuperSignal substrate and incubated for 1 min. The primary and secondary antibodies used are detailed in Supplementary Table 2.

### 2.7 Statistical analysis

Quantitative data are expressed as the mean ± standard deviation of at least three replicates per experiment. Statistical analysis was performed using One-way ANOVA by GraphPad Prism (GraphPad Software, Inc., USA). p < 0.05 was considered to be significant. The levels of significance were set at *p < 0.05, **p < 0.01, ***p < 0.001, and ****p < 0.0001.

## 3 Results

### 3.1 Extracellular P_i_ down-regulates the expression of *ALPL* and *PHOSPHO1* in human osteoblasts via type III Na-Pi co-transporters

**Fig. 1:**
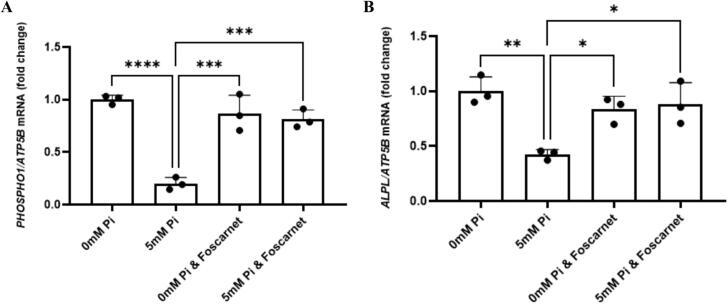
The down regulation of human osteoblast PHOSPHO1 and ALPL expression by extracellular P_i_ is mediated by PiT1/Pit2. (A) PHOSPHO1 and (B) ALPL expression is down regulated by 5 mM P_i_ after 24 h of culture. Treatment with Foscarnet (300 μM) completely prevented the down-regulation of *PHOSPHO1* and *ALPL* expression by 5 mM P_i_. The cells were maintained in P_i_-free DMEM supplemented with 10 % FBS until confluent and thereafter for a further 10 days in P_i_-free DMEM supplemented with 10 % FBS, l-ascorbic acid and CaCl₂ to promote the differentiated phenotype prior to P_i_ challenge for 24 h. Statistical significance is denoted as follows: *p < 0.05, **p < 0.01, ***p < 0.001, ****p < 0.0001. Data represents mean ± SD, with three replicates per group.

Previous studies using primary murine osteoblasts and osteoblast cell lines have shown that extracellular P_i_ can alter the expression of osteoblast genes via the type III Na/P_i_ co-transporters, PiT1 and PiT2 (Beck Jr. and Knecht, 2003; Beck Jr. et al., 2000; Bon et al., 2018a). We therefore first determined if extracellular P_i_ was also able to control *ALPL* and *PHOSPHO1* expression by human primary osteoblasts and if this regulation was mediated by PiT1 and/or PiT2. Osteoblasts treated for 24 h with 5 mM P_i_ expressed reduced levels of both *PHOSPHO1* and *ALPL* when compared to osteoblasts cultured in Pi-free medium (Fig. 1A, B). Treatment with foscarnet prevented the decrease in *PHOSPHO1* and *ALPL* expression induced by elevated extracellular P_i_ suggesting that the inhibitory effect of P_i_ on the expression of these genes is mediated through type III Na-Pi co-transporters.

### 3.2 Extracellular P_i_ down regulates *PHOSPHO1* and *ALPL* expression in human osteoblasts via ERK1/2 phosphorylation

**Fig. 2:**
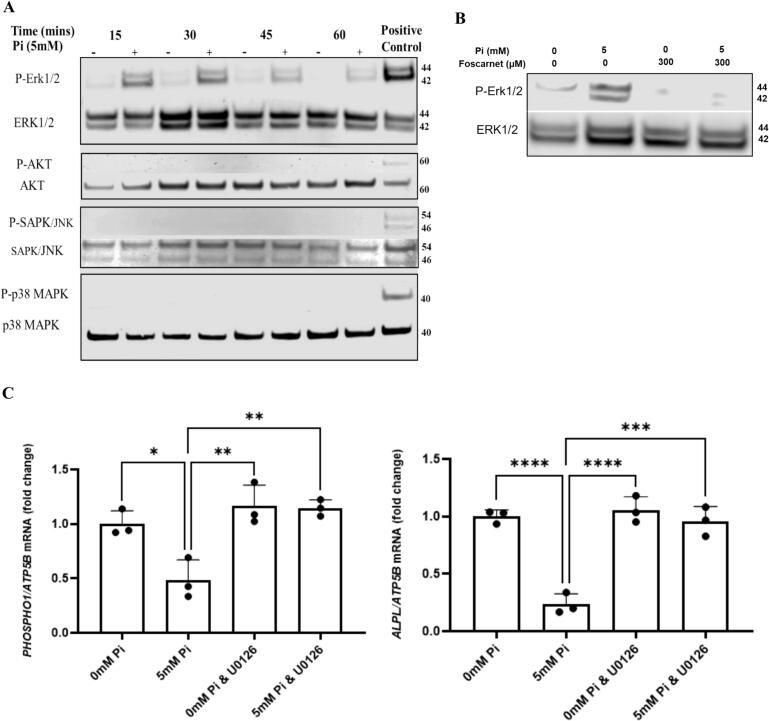
Extracellular P_i_ down regulates *PHOSPHO1* and *ALPL* expression via ERK1/2 phosphorylation (A) Western blots showing total and phosphorylated levels of ERK1/2, AKT, SPAK/JNK, and P38/MAPK proteins in osteoblasts treated with 0 or 5 mM P_i_ for 0, 15, 30, and 45 min. Only ERK1/2 was phosphorylated by 5 mM P_i_ and this was seen at all time points studied. Insulin (100 nM) was used as a positive control for Erk1/2 and Akt activation whereas anisomycin (2.5 μg/mL) was used as a positive control for SAPK/JNK and p38MAPK activation. (B) ERK1/2 phosphorylation by treatment with 5 mM P_i_ (30 min) was inhibited by co-treatment with Foscarnet. (C) Inhibition of ERK1/2 phosphorylation by UO126 blocked the ability of 5 mM P_i_ to reduce the expression of *PHOSPHO1* and *ALPL.* The cells were maintained in P_i_-free DMEM supplemented with 10 % FBS until confluent and for the cell signalling studies (A, B) the cells were serum starved overnight before challenged with fresh medium containing 5 mM P_i_ for up to 60 min. For the gene expression studies (C) the confluent cultures were cultured for a further 10 days in P_i_-free DMEM supplemented with 10 % FBS, l-ascorbic acid and CaCl₂ to promote the differentiated phenotype prior to P_i_ challenge for 24 h. All inhibitors were introduced to the cultures 60 min prior to the addition of P_i_. Statistical significance is denoted as follows: *p < 0.05, **p < 0.01, ***p < 0.001, ****p < 0.0001. Data represents mean ± SD, with three replicates per group.

We next identified the intracellular signalling pathway(s) utilised by P_i_ to down regulate *PHOSPHO1* and *ALPL* expression. Western blot analysis revealed that ERK1/2 but not AKT, SPAK/JNK or p38 MAPK was phosphorylated after 15-, 30-, 45- and 60-min treatment with 5 mM Pi (Fig. 2A). The activation of ERK1/2 by 5 mM P_i_ was blocked by co-treatment with foscarnet and inhibition of ERK1/2 activation by UO126 prevented the decrease in *PHOSPHO1* and *ALPL* expression induced by 24 h treatment with 5 mM Pi (Fig. 2B, C). These data indicate that extracellular P_i_ requires ERK1/2 activation to down regulate the expression of *ALPL* and *PHOSPHO1* by osteoblasts.

### 3.3 Down regulation of *PHOSPHO1* and *ALPL* expression by extracellular P_i_ in human osteoblasts requires FGFR signalling

**Fig. 3:**
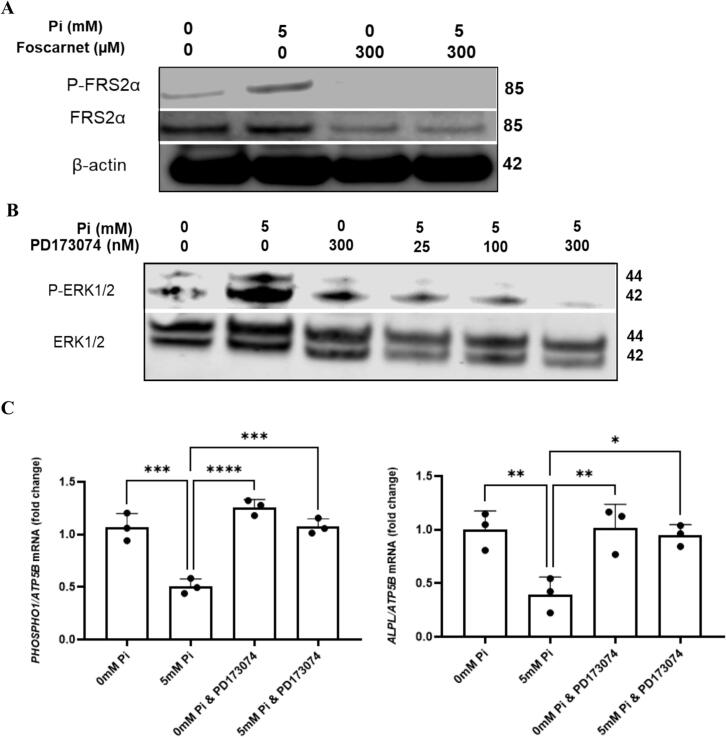
Extracellular P_i_ down regulates *PHOSPHO1* and *ALPL* expression via PiT1/PiT2–FGFR crosstalk (A) Foscarnet blocked the increase in osteoblast FRS2α phosphorylation by 5 mM P_i_ (30 min). (B) FGFR signalling inhibition by PD173074 blocked ERK 1/2 phosphorylation by 5 mM P_i_ (30 min) (C) Inhibition of FGFR signalling by PD173074 blocked the ability of 5 mM P_i_ to reduce the expression of *ALPL* and *PHOSPHO1* after 24 h of culture*.* The cells were maintained in P_i_-free DMEM supplemented with 10 % FBS until confluent and for the cell signalling studies (A, B) the cells were serum starved overnight before challenged with fresh medium containing 5 mM P_i_ for up to 60 min. For the gene expression studies (C) the confluent cultures were cultured for a further 10 days in P_i_-free DMEM supplemented with 10 % FBS, l-ascorbic acid and CaCl₂ to promote the differentiated phenotype prior to P_i_ challenge for 24 h. All inhibitors were introduced to the cultures 60 min prior to the addition of P_i_. Statistical significance is denoted as follows: *p < 0.05, **p < 0.01, ***p < 0.001, ****p < 0.0001. Data represents mean ± SD, with three replicates per group.

Some studies have reported that P_i_ control of gene expression in osteoblasts and chondrocytes requires cross-talk between type III Na/P_i_ co-transporters and FGFR signalling (Kimata et al., 2010; Nishino et al., 2017; Takashi et al., 2019). We examined this in human osteoblasts and found that 5 mM P_i_ phosphorylated FRS2α (downstream of FGFR signalling) and this activation was abolished by foscarnet (Fig. 3A). Foscarnet also reduced the expression of total FRS2α but equal loading of the membrane was confirmed by β-actin expression (Fig. 3A). Also, blocking FGFR signalling by PD173074 inhibited the phosphorylation of ERK1/2 by 5 mM P_i_ (Fig. 3B). Similarly, PD173074 prevented the decrease in *ALPL* and *PHOSPHO1* expression after 24 h treatment with 5 mM P_i_ (Fig. 3C).

### 3.4 Human osteoblast matrix mineralisation is controlled by P_i_ uptake via type III Na-Pi co-transporters and FGFR signalling

**Fig. 4:**
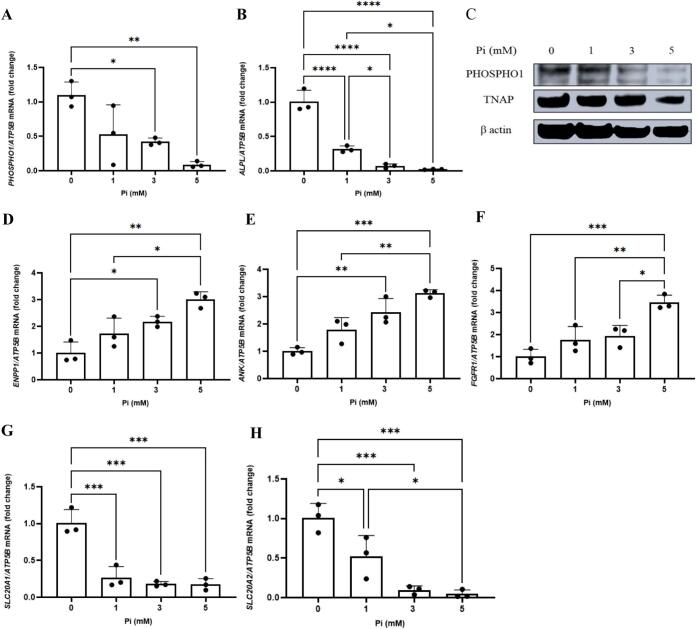
Extracellular P_i_ alters the expression of bone matrix mineralisation genes in long term cultures of human osteoblasts (A, B) *PHOSPHO1* and *ALPL* expression are both down regulated with increasing extracellular P_i_ concentrations. (C) PHOSPHO1 and TNAP protein expression was decreased with increasing concentrations of P_i_. (D–F) Expression of *ENPP1, ANK* and *FGFR1* are all positively correlated to extracellular P_i_ concentrations. (G, H) Expression of SLC20A1 and SLC20A2 are both negatively correlated to extracellular P_i_ concentrations. The cells were maintained in P_i_-free DMEM supplemented with 10 % FBS until confluent after which they were cultured for a further for 2-weeks in P_i_ -free DMEM supplemented with ascorbic acid, CaCl_2_ together with varying concentrations of P_i._ Statistical significance is denoted as follows: *p < 0.05, **p < 0.01, ***p < 0.001, ****p < 0.0001. Data represents mean ± SD, with three replicates per group.

**Fig. 5:**
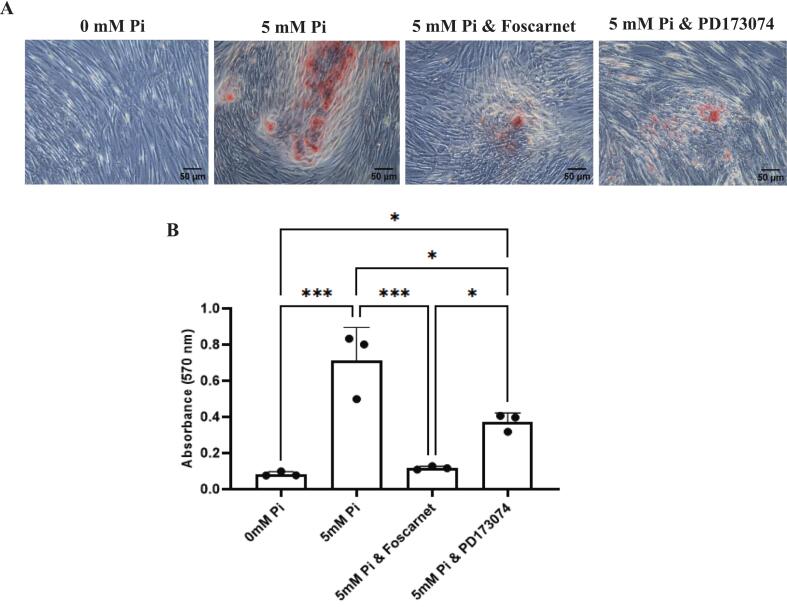
Foscarnet and PD173074 inhibit the effects of extracellular P_i_ on osteoblast matrix mineralisation. A: Representative images of Alizarin red S staining in response to 0 mM P_i_, 5 mM P_i_, 5 mM Pi & Foscarnet and 5 mM P_i_ & PD173074 after 2-weeks' post-confluency culture. B: Quantification of Alizarin red S staining. The cells were maintained in P_i_-free DMEM supplemented with 10 % FBS until confluent and to induce mineralisation they were cultured for a further for 2-weeks in P_i_ -free DMEM supplemented with ascorbic acid, CaCl_2_ together with 5 mM P_i_ and inhibitors. Both inhibitors were introduced to the cultures 60 min prior to the addition of P_i_. Data represents mean ± SD, with three replicates per group.

The studies described so far have reported the expression levels of *ALPL* and *PHOSPHO1* by osteoblasts after a 24 h challenge with extracellular P_i_. To more closely mimic the duration period of the mineralisation process, we next determined the longer-term effects of extracellular P_i_ on *ALPL* and *PHOSPHO1* expression and also the mineralisation process itself. Osteoblasts continuously cultured (without expansion) in variable concentrations of extracellular P_i_ (0, 1, 3, 5 mM) for 2-weeks resulted in a concentration dependent decrease in both *ALPL* and *PHOSPHO1* expression. A similar trend was also noted at the protein level (Fig. 4A–C). In addition, we also determined the expression levels of *ENPP1* and *ANK* as they are major regulators of matrix mineralisation through their role in controlling the production of extracellular pyrophosphate, a potent inhibitor of mineralisation (Meyer, 1984). We found a positive correlation between P_i_ concentrations and both *ENPP1* and *ANK* expression (Fig. 4D, E). A positive correlation between P_i_ concentration and *FGFR1* expression was also observed but, in contrast, the expression of both SLC20A1 (encodes Pit1) and SLC20A2 (encodes Pit2) was decreased with higher P_i_ concentrations (Fig. 4F–H). Matrix mineralisation was enhanced by 5 mM Pi and this was dependent upon type III Na- P_i_ co-transporters and FGFR signalling (Fig. 5).

### 3.5 Extracellular P_i_ down-regulates the expression of *Alpl* and *Phospho1* in murine calvariae via type III Na-P_i_ co-transporters and FGFR signalling

**Fig. 6:**
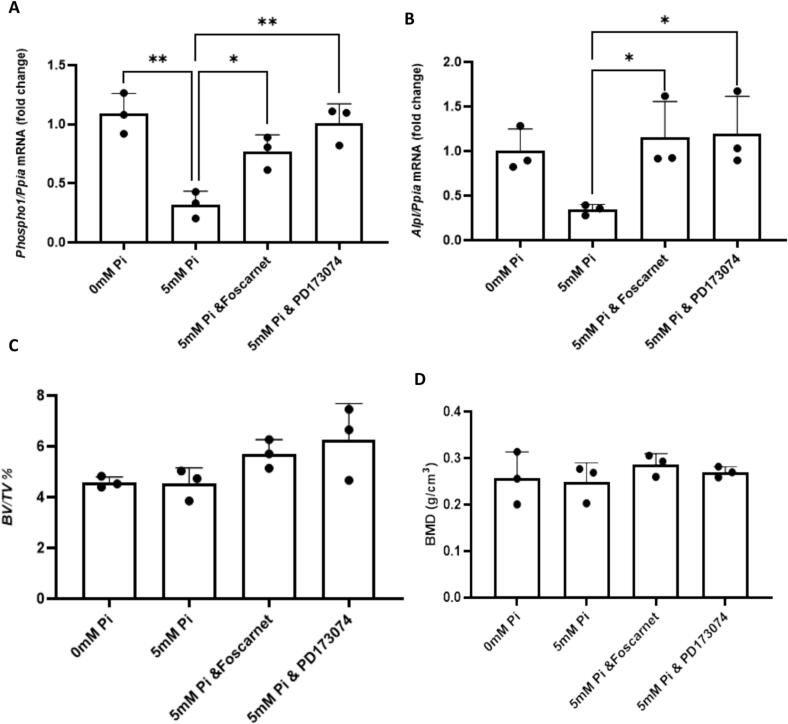
Extracellular P_i_ down regulates murine calvaria *Phospho1* and *Alpl* expression via Pit1/Pit2–FGFR crosstalk but has no effect on calvaria bone mass and bone mineral density. Exposure to 5 mM Pi for 3-days in culture resulted in a reduction of (A) *Phospho1* and (B) *Alpl* expression which was prevented by co-incubation with Foscarnet or PD173074. Exposure to 5 mM P_i_ with or without Foscarnet or PD173074 had no effect on BV/TV or BMD (C, D). The half-calvaria were cultured overnight in P_i_-free DMEM supplemented with 10 % FBS and thereafter fresh medium supplemented with ascorbic acid, CaCl₂ together with varying concentrations of P_i_ and inhibitors for 3-days. The calvariae were pre-incubated with foscarnet or PD173074 for 60 min prior to the addition of P_i_. Statistical significance is denoted as follows: *p < 0.05, **p < 0.01. Data represents mean ± SD, with three replicates per group.

To translate the cell culture results to a more physiological mineralisation model we next determined if extracellular Pi could down regulate *Alpl* and *Phospho1* expression in murine calvaria and mimic what was observed in human primary osteoblasts (Fig. 1). As observed in human osteoblasts the expression of both phosphatases was down regulated by 5 mM P_i_ and this was mediated by type III Na- P_i_ co-transporters and FGFR signalling (Fig. 6A, B). Despite changes in phosphatase expression none of the treatments were able to affect bone volume or BMD (Fig. 6C, D).

## 4 Discussion

Bone is a two-phase composite material made up of mostly organic collagen type I and carbonate substituted hydroxyapatite mineral (Boskey, 2013). The formation of hydroxyapatite is dependent upon the availability of calcium and P_i_ to complex together and interact with collagen to form bone that is rigid, tough and resistant to fracture (Boskey, 2003; Posner, 1985). The inability to maintain serum P_i_ concentrations in the normal range results in soft bones (phosphopenic rickets) but hypophosphatemia (<2.5 mg/dL) is rarely a result of nutritional P_i_ deficiency. Rather, it is a consequence of either hypocalcaemia and secondary hyperparathyroidism, increased renal P_i_ wasting as a result of mutations in the PHEX gene (phosphate-regulating gene with homologies to endopeptidase on the X chromosome) or reduced absorption due to impaired vitamin D actions (Carpenter et al., 2017).

Extracellular fluid is normally supersaturated with calcium and P_i_ but when deficient in either, it is likely that mineral nucleation within the bone matrix will be stalled. While the site at which mineral is initially deposited and the mechanisms that regulate its deposition are unclear, there is a body of evidence to support a role for matrix vesicles (MVs) in the biomineralisation process. To generate and control the local concentrations of P_i_ to promote mineralisation, MVs require both TNAP and PHOSPHO1 (Dillon et al., 2019; Millán, 2013). Both phosphatases are expressed by osteoblasts and are present within MVs (Ali et al., 1970; Anderson, 1995; Roberts et al., 2007) and when either is deficient in mice (Anderson et al., 2004; Fedde et al., 1999; Huesa et al., 2011; Yadav et al., 2011) or enzyme activity is inhibited in the developing chick or osteoblast cultures (Huesa et al., 2015; Staines et al., 2021) there is an impairment of matrix mineralisation. While PHOSPHO1 and TNAP have non-redundant roles in the mineralisation process their joint function is required to avoid defective skeletal mineralisation (Huesa et al., 2015; Macrae et al., 2010; Yadav et al., 2011). Furthermore, the defective skeletal and dental mineralisation in patients with hereditary hypophosphatasia and the atypical femur fractures noted in humans with PHOSPHO1 mutations phenocopy the skeletal defects reported in *Alpl* and *Phospho1* deficient mice (Marini et al., 2023; Weiss et al., 1988). Despite the availability of systemic P_i_ in mice deficient in both *Phospho1* and *Alpl* they show perinatal lethality and a complete lack of skeletal mineralisation (Yadav et al., 2011). As reported previously, this suggests that organic P_i_, such as ATP or ADP, may be used by TNAP for the local generation of P_i_ (Yadav et al., 2011). This would concur with the results of Yoshiko and colleagues who proposed that osteoblasts control skeletal mineralisation via an endogenous P_i_-sensing system that is independent of systemic P_i_ homeostasis (Yoshiko et al., 2007).

While these observations strongly support the need for locally produced P_i_ in the initiation of matrix mineralisation the relationship, if any, between extracellular P_i_ concentrations and osteoblast phosphatase expression is unclear and was the focus of this study. Here, we report that human primary osteoblasts exposed to extracellular P_i_ for 24 h decreased their expression of both *PHOSPHO1* and *ALPL* via PiT1/Pit2 sodium-P_i_ co-transporters and the ERK 1/2 signalling pathway. A similar *Alpl* response to extracellular P_i_ by mouse ATDC5 chondrocyte-like cells and the mouse ST2 stromal cells has been reported (Goseki-Sone et al., 1999; Kimata et al., 2010). Nevertheless, other studies with mouse primary osteoblasts, human osteoblast like-cells and mesenchymal stem cells show inconsistencies in the *ALPL* response to extracellular Pi (Orimo and Shimada, 2006; Ren et al., 2023; Rendenbach et al., 2014). The requirement for PiT1/PiT2 transporters and ERK 1/2 signalling for the control of P_i_ responsive genes such as *Spp1*, *Mgp*, *Dmp1*, *Postn*, *Dcn* and *Ogn* is reported in studies using mouse MC3T3 (Beck Jr. and Knecht, 2003; Beck Jr. et al., 2000; Bon et al., 2018a; Camalier et al., 2013; Conrads et al., 2005; Julien et al., 2009) and rat UMR106 (Takashi et al., 2019) osteoblast-like cells, mouse ATDC5 chondrogenic-like cells (Julien et al., 2007; Kimata et al., 2010), and primary mouse osteoblasts and osteocytes (Julien et al., 2009; Nishino et al., 2017). The ability of extracellular P_i_ to regulate the expression of matrix proteins, and promotors and inhibitors of mineralisation suggests that P_i_ can transform the matrix to a mineralisation competent state.

Foscarnet does not discriminate between PiT1 and PiT2 but studies with MC3T3 osteoblast-like cells indicate that silencing either co-transporter blunted the ability of P_i_ to activate ERK 1/2 and up-regulate the expression of *Mgp* and *Ocn* (Bon et al., 2018a). The altered *Mgp* and *Ocn* expression was despite normal P_i_ uptake by the cell casting doubt on the dependency of variation in intracellular P_i_ content as the trigger for altered gene expression (Bon et al., 2018a). Indeed, data from cells over-expressing transport deficient PiT1 and PiT2 mutants inferred that P_i_ sensing rather than uptake by the cell is required for P_i_ signalling through the PiT proteins (Beck et al., 2009; Bon et al., 2018a; Chavkin et al., 2015).

Our data also suggests that the phosphorylation of ERK 1/2 and the down regulation of osteoblast *ALPL* and *PHOSPHO1* expression by P_i_ involves PiT1/PiT2 – FGFR crosstalk. Although the involvement of FRS2α in P_i_ initiated signalling has been reported by others (Camalier et al., 2013; Kimata et al., 2010; Nishino et al., 2017; Suzuki et al., 2000; Takashi et al., 2019), only limited insight exists to explain the FGFR role in mediating PiT signal transduction triggered by increased extracellular P_i_. The stimulation of N-Ras and activation of AP-1 may be involved and Yamasaki and colleagues disclosed that overexpression of FGFR1 is able to restore P_i_ responsiveness to cells in which *Slc20a1* was silenced (Camalier et al., 2013; Yamazaki et al., 2010). Moreover, the tyrosine residues of FRS2α phosphorylated by extracellular P_i_ and FGF2, a canonical FGFR ligand, are different (Yamazaki et al., 2010). The intimate relationship between P_i_ metabolism and PiT/FGF/FGFR signalling may have broader implications for diseases with altered systemic P_i_ levels such as chronic kidney disease and x-linked hypophosphatemic rickets that are characterised by increased FGF23 serum levels (Bon et al., 2018b; Kinoshita and Fukumoto, 2018; Wolf, 2012).

A decrease in PHOSPHO1 and ALPL expression by extracellular P_i_ (1 mM and higher) is also noted in our longer-term osteoblast and calvaria cultures that were designed to directly examine the interplay between exogenous P_i_ and locally generated P_i_ in the control of matrix mineralisation. A role for calcium phosphate precipitates in reducing phosphatase expression cannot be ruled out but as precipitates are unlikely to form in the presence of 1 mM P_i_ it is doubtful that sensing mechanisms in this case involve extracellular crystals (Khoshniat et al., 2011). Despite lower phosphatase expression, matrix mineralisation was increased in osteoblast cultures, but not murine calvaria, treated with 5 mM P_i_. This is in agreement with earlier studies using murine primary osteoblasts and may suggest that when exogenous P_i_ is plentiful to promote matrix mineralisation there is no requirement to generate Pi locally (Hsu et al., 2022). Furthermore, the promotion of osteoblast matrix mineralisation by extracellular P_i_ occurred in spite of increased *ANK* and *ENPP1* expression, whose gene product is responsible for regulating P P_i_ levels within the extracellular matrix. Similar increases in *Ank* and *Enpp1* expression by mouse osteoblasts by extracellular P_i_ have been previously noted (Rendenbach et al., 2014) and may, along with decreased local phosphatase expression, contribute to the accumulation of unmineralised osteoid observed in Fgf23-deficient mice, despite a marked hyperphosphatemia (Sitara et al., 2004). Furthermore, as foscarnet shares a similar structure to PP_i_ it may have a direct role on mineralisation (Fleisch et al., 1970). However, the inhibitory effect of foscarnet on the mineralisation ability of the ATDC5 chondrocyte cell line was only noted at concentrations of 5 mM and therefore a direct inhibitory effect of 300 μM foscarnet on mineralisation as used in this study is unlikely (personal communication – Monzur Murshed, McGill University, Canada). It is not evident why extracellular P_i_ was unable to modify calvaria bone volume but the length of culture may have been insufficient to detect changes. In a similar study, BV/TV of cultured calvaria was increased in P_i_ containing osteogenic medium after 9- but not 2-days of culture (Zheng et al., 2020). However, in a study by Mundy and colleagues new bone formation in postnatal murine calvaria culture was reported after 3-days in culture (Mundy et al., 1999). We speculated that the mineralisation of this new bone would be influenced by the presence of extracellular Pi ± foscarnet or PD173074. The reason we did not see this is unclear but in postnatal calvaria, unlike osteoblasts in cultures, matrix mineralisation is already present. Once started, mineralisation may proceed without exogenous P_i_ and this may explain the lack of effect of the various treatments on BMD in calvaria explants (Bellows et al., 1992).

Nevertheless, in isolated osteoblasts the promotion of matrix mineralisation by exogenous P_i_ was blunted in the presence of FGFR or type III Na-Pi co-transporter inhibitors, suggesting that Pi sensing and/or uptake by the cell is required for the control of matrix mineralisation (Yoshiko et al., 2007). Of interest, P_i_ is also able to stimulate MV release from the parent cell (Chaudhary et al., 2016). The accumulation of P_i_ within osteoblasts to mediate matrix mineralisation is likely to be facilitated by PiT-1 and PiT-2 but intriguingly the expression of both *SLC20A1* and *SLC20A2* is decreased by exogenous P_i_, an observation that is at odds with similar studies in osteoblast, chondrocyte and odontoblast cell lines (Beck Jr. et al., 2003; Wittrant et al., 2009; Zoidis et al., 2004). The uptake of P_i_ into the lumen of MVs is also likely, but the identity of the transporter involved is unclear. Phosphate transporters have been identified in MVs (Montessuit et al., 1995, Montessuit et al., 1991) however several proteomic studies have failed to detect PiT-1, PiT-2, nor any other sodium-coupled phosphate transporter within MVs (Balcerzak et al., 2008; Xiao et al., 2007). Notwithstanding, we have previously reported that mice deficient in both PHOSPHO1 and PiT-1 have a skeleton that is more hypomineralised than that noted in PHOSPHO1 KO mice (Yadav et al., 2016).

The ability of phosphatases to respond to Pi availability is not restricted to cells regulating matrix mineralisation. PHOSPHO1 is a member of the haloacid dehalogenase (HAD) superfamily of phosphatases (Houston et al., 1999) and shares ~30 % homology at the amino acid level with a tomato phosphate starvation-induced gene product, LePS2, whose expression is induced in the absence, but repressed in the presence, of P_i_ (Baldwin et al., 2001; Stenzel et al., 2003). While, LePS2 was the first P_i_ starvation-induced HAD-like acid phosphatase to be identified in plants others have been subsequently found. These include a Ser/Thr phosphatase translated by the PvHAD1 gene in the common bean, *Phaseolus vulgaris* L. and OsHAD2 in the rice, *Oryza sativa* that belongs to the PHOSPHO1 subfamily (Burroughs et al., 2006; Du et al., 2021; Hur et al., 2007; Liu et al., 2010) Furthermore, similar to PHOSPHO1, AtPECP1 and AtPECP2 are reported to hydrolyse PCho and PEA under P_i_ starvation conditions in Arabidopsis (Angkawijaya and Nakamura, 2017; Hanchi et al., 2018) The existence of numerous P_i_ starvation-induced HAD-like acid phosphatase in plants suggests an ability to adapt to P_i_-deficient conditions and a similar fail-safe system arrangement may operate in vertebrates in an attempt to protect the biomineralisation process when systemic and extracellular Pi is limited.

In conclusion, altered expression of phosphatases by osteoblasts in response to changing extracellular P_i_ concentrations supports the premise that osteoblasts are able to regulate the biomineralisation of bone via a P_i_-sensing mechanism. This regulatory process is likely to have implications for pathological conditions associated with serum P_i_ excess or deficiency. These include chronic kidney disease associated osteomalacia and hypophosphatemic rickets. An ability to manipulate P_i_ sensing, akin to what has been done with the calcium sensing receptor, may offer targeted therapies to combat mineralisation defects observed in various metabolic bone disorders.

## CRediT authorship contribution statement

**Soher Nagi Jayash:** Writing – review & editing, Writing – original draft, Visualization, Validation, Supervision, Software, Methodology, Investigation, Formal analysis, Data curation, Conceptualization. **Thomas Duff:** Writing – review & editing, Investigation, Formal analysis. **Qaisar Tanveer:** Writing – review & editing, Methodology, Investigation, Formal analysis. **Worachet Promruk:** Writing – review & editing, Software, Investigation, Formal analysis. **Colin Farquharson:** Writing – review & editing, Writing – original draft, Validation, Supervision, Project administration, Methodology, Funding acquisition, Formal analysis, Data curation, Conceptualization.

## Declaration of competing interest

The authors declare the following financial interests/personal relationships which may be considered as potential competing interests: Qaisar Tanveer reports financial support was provided by The Punjab Educational Endowment Fund (PEEF), Government of Punjab, Pakistan. If there are other authors, they declare that they have no known competing financial interests or personal relationships that could have appeared to influence the work reported in this paper.

## Data Availability

All data are available in the manuscript.

## Appendix A Supplementary data

Supplementary tables Image 1
