# Supplementary material for: Osteoblasts sense extracellular levels of phosphate to control the local expression of phosphatases for matrix mineralisation
Source: Bone Rep. 2025 Jul 30;26:101863. doi: 10.1016/j.bonr.2025.101863 (PMC12337689; doi:10.1016/j.bonr.2025.101863)
Supplement: Supplementary file 1 — Supplementary tables [file mmc1.docx]

**Supplementary table 1:** Primer sequences for qPCR

| **Genes** | **Forward, 5’ -> 3’** | **Reverse, 5’ -> 3’** | **Source** |
| --- | --- | --- | --- |
| Human PiT1 (*SLC20A1*)_ | CTGCCGAGTGTCTACCAGTC | TGTTGCTGTGTTTCCCAACG | Sigma-Aldrich |
| Human PiT2 (*SLC20A2*) | TCTTCGTGTGTCCGTGGATG | TCATTAGCCTTGGCACCTGG |  |
| Human *PHOSPHO1* | TCTCCGATGCCAACACCTTT | GTAGAAGAGGCGCTCGAAGT |  |
| Human *ALPL* | GGCACCTGCCTTACTAACTCC | TGTGGAGACACCCATCCCAT |  |
| Human *ATP5B* | TCTTGTGCAGCCTACACAGAAAA | AGTGGTGAGGAATGTTACATGATGC | Invitrogen |
| Human ENPP1 | ATGTCGTCAGTGGTCCTGTG | CTGTGATCCGTGCTCTGTGT | Thermo Fisher Scientific  Thermo Fisher Scientific  Thermo Fisher Scientific |
| Human  ANK | ACATAGCCATCGACTTCGGG | TCAGTGTGTGAAAGACGGCA |  |
| Human FGFR1 | AGAGCGGGGAGTATGTGTGT | TCTCTTCCAGGGCTTTTGCC |  |
| Mice  Phospho1 | TTCTCATTTCGGATGCCAACA | TGAGGATGCGGCGGAATAA |  |
| Mice  Alpl | GGGACGAATCTCAGGGTACA | AGTAACTGGGGTCTCTCTC |  |
| Mice  Ppia | CACCGTGTTCTTCGACATCA | CAGTGCTCAGAGCTCGAAAGT | Sigma-Aldrich |

**Supplementary Table 2:** Antibodies for western blotting

| **Type** | **Target** | **Source** | **Dilution** | **Company** | **Molecular weight** |
| --- | --- | --- | --- | --- | --- |
| Primary | FRS2α | Mouse | 1:500 | Santa Cruz Biotechnology, US | 85 |
|  | P-FRS2α | Rabbit |  | Cell Signalling, US | 85 |
|  | ERK1/2 | Rabbit | 1:1000 |  | 42-44 |
|  | P-ERK1/2 | Rabbit |  |  | 42-44 |
|  | AKT | Rabbit |  |  | 60 |
|  | P- AKT | Rabbit |  |  | 60 |
|  | JNK | Rabbit |  |  | 46-54 |
|  | P- JNK | Rabbit |  |  | 46-54 |
|  | p38/ MAPK | Rabbit |  |  | 40 |
|  | P-p38/ MAPK | Rabbit |  |  | 40 |
|  | PHOSPHO1 | Human | 1:500 | AbD Serotec and Bio-Rad | 32 |
|  | TNAP | Rat | 1:1000 | R&D Systems | 75 |
|  | β-actin | Rabbit | 1:5000 | Cell Signalling Technology | 42 |
|  | **Source** | | **Dilution** | **Company** | |
| Secondary | Donkey anti-mouse | | 1:5000 | LI-COR, US | |
|  | Donkey anti-rabbit | |  |  |  |
|  | Goat anti-rabbit for ECL | | 1:1000 | Dako | |
|  | Goat anti-human for ECL | | 1:500 | Bio-Rad | |
|  | Goat anti-rat for ECL | | 1:1000 | R&D Systems | |
